# Comparative Study of Augmented Classical Least Squares Models for UV Assay of Co-Formulated Antiemetics Together with Related Impurities

**DOI:** 10.3390/molecules28207044

**Published:** 2023-10-12

**Authors:** Muneera S. M. Al-Saleem, Hany W. Darwish, Ibrahim A. Naguib, Mohammed E. Draz

**Affiliations:** 1 Department of Chemistry, Science College, Princess Nourah bint Abdulrahman University, P.O. Box 84428, Riyadh 11671, Saudi Arabia; msalsaleem@pnu.edu.sa; 2 Department of Pharmaceutical Chemistry, College of Pharmacy, King Saud University, P.O. Box 2457, Riyadh 11451, Saudi Arabia; hdarwish@ksu.edu.sa; 3 Department of Pharmaceutical Chemistry, College of Pharmacy, Taif University, P.O. Box 11099, Taif 21944, Saudi Arabia; 4 Department of Pharmaceutical Chemistry, Faculty of Pharmacy, Delta University for Science and Technology, Gamasa 35712, Egypt; mohamadderaz9999@gmail.com

**Keywords:** classical least squares, training set, independent test set, NAP/CLS, OSC/CLS, DOSC/CLS

## Abstract

The classical least squares (CLS) model and three augmented CLS models are adopted and validated for the analysis of pyridoxine HCl (PYR), cyclizine HCl (CYC), and meclizine HCl (MEC) in a quinary mixture with two related impurities: the CYC main impurity, Benzhydrol (BEH), which has carcinogenic and hepatotoxic effects, and the MEC official impurity, 4-Chlorobenzophenone (BEP). The proposed augmented CLS models are orthogonal signal correction CLS (OSC-CLS), direct orthogonal signal correction CLS (DOSC-CLS), and net analyte processing CLS (NAP-CLS). These models were applied to quantify the three active constituents in their raw materials and their corresponding dosage forms using their UV spectra. To evaluate the CLS-based models sensibly, we design a comparative study involving two sets: the training set to construct models and the validation set to assess the prediction abilities of these models. A five-level, five-factor calibration design was established to produce 25 mixtures for the calibration set. In addition, 16 experiments were performed for a test set distributed equally between the in-space and out-space samples. The primary criterion for comparing the models’ performance was the validation set’s root mean square error of prediction (RMSEP) value. Finally, augmented CLS models showed acceptable results for assaying the three analytes. The results were compared statistically with the reported HPLC methods; however, the DOSC-CLS model proved the best for assaying the dosage forms.

## 1. Introduction

The pharmaceutical industry needs to develop new analytical methods to resolve complex mixtures. Analysts are continually driven to devise approaches that are not only economical and environmentally friendly but also robust and efficient. The application of chemometric techniques to interpret complex UV spectra has emerged as an intelligent solution, enabling the simultaneous analysis of multiple drugs within complex mixtures. In light of this, our study aims to shed light on the comparative strengths and limitations of four distinct chemometric models: the classical least squares (CLS) model, orthogonal signal correction CLS (OSC-CLS), direct orthogonal signal correction CLS (DOSC-CLS), and net analyte processing CLS (NAP-CLS). By conducting this comprehensive comparison, we seek to highlight the unique attributes and potential shortcomings of each model, providing valuable insights for analytical chemists and researchers in the pharmaceutical field.

**Figure 1:**
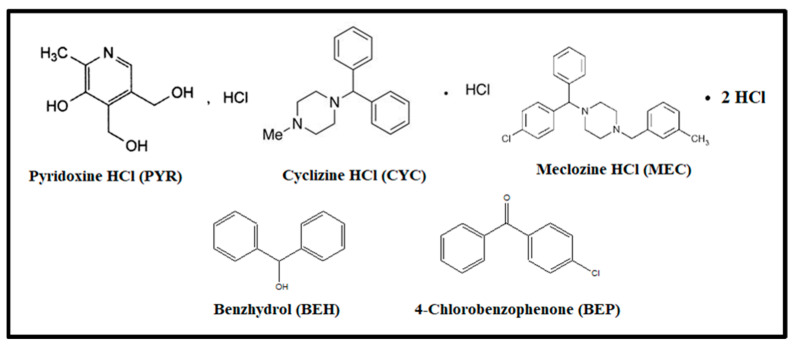
Chemical structures of pyridoxine HCl, cyclizine HCl, Meclozine HCl, Benzhydrol, and 4-Chlorobenzophenone.

The UV dataset of a quinary mixture is used as a case study. The mixture under investigation is composed of three active constituents, namely pyridoxine HCl (PYR) (known as vitamin B6), cyclizine (CYC), and meclizine HCl (MEC), in addition to two related impurities called Benzhydrol (BEH) and 4-Chlorobenzophenone (BEP). British Pharmacopoeia [1] chemically identifies the studied drugs as follows: PYR, 5-hydroxy-6-methyl pyridine-3,4-diyl di methanol hydrochloride; CYC, 1-benzhydryl-4-methylpiperazine; MEC, 1-[(R.S.)-(4-chlorophenyl) phenylmethyl]-4-[(3-methyl phenyl)methyl] piperazine dihydrochloride; BEH, diphenylmethanol; and BEP, (4-chlorophenyl) phenyl methanone (Figure 1). PYR is the drug of choice for treating nausea and vomiting with an unrecognized mechanism of action [1,2], while CYC and MEC exert their antiemetic effects through the antagonism of 1H receptors [3]. PYR was formulated with MEC or CYC in many pharmaceutical products to synergize the antiemetic activity [2]. Most pregnant women usually suffer from hyperemesis gravidarum syndrome in their first months of pregnancy [3]. This syndrome, characterized by nausea and vomiting, may lead to dehydration and weight loss in certain complications [2]. The pregnant women used combined pharmaceutical products to inhibit this syndrome. Regarding the related impurities, the first one is BEH, which is considered the CYC main impurity [1]. This impurity has proved to be carcinogenic and possesses a hepatotoxic effect [4,5,6], which adds value to the presented study. The other impurity, BEP, is the official impurity of MEC [1].

The literature search revealed the analysis of studied drugs in binary or ternary mixtures as follows: binary mixtures of PYR with either CYC or MEC were determined by HPLC methods [7,8,9], spectrophotometric methods [7,8,10,11,12], and UV chemometric methods [7,8]. The ternary mixture was presented for analysis in two studies. The first one implemented the TLC method [13], while the other implemented spectrophotometric, UPLC, and chemometric methods [14]. In a previous study of our team members [15], the quintuple mixture was analyzed by the partial least squares (PLS) method and support vector regression (SVR) methods; however, SVR is well known for complicated optimization steps, and PLS loses qualitative information about mixture components during regression steps. Accordingly, more straightforward chemometric methods would be of high value to introduce.

The work presented here aims to achieve two primary goals. First, it makes a simple comparison between the aforementioned CLS-based chemometric models—in an attempt to demonstrate their flaws and features—using the UV dataset of the quinary mixture. The related impurities make the comparison challenging due to their structural similarities to the main drugs and the significant difference between their amounts relative to the active constituents [16]. Accordingly, the evolved analytical methods should have specific selectivity [16]. In the field of analytical chemistry, various modeling techniques have been employed to address the challenges posed by complex mixtures of pharmaceutical compounds. CLS is a well-established method that has played a significant role in analytical chemistry for decades. It relies on the principles of linear regression and is particularly useful when dealing with spectral data [17].

While CLS has been a cornerstone in analytical chemistry, modern advancements have introduced alternative techniques such as PLS and deep-learning methods. PLS, for example, offers advantages in situations where collinearity among variables is a concern and has become a popular choice for multivariate analysis in spectroscopy. Deep-learning techniques, on the other hand, leverage neural networks to automatically extract intricate patterns from complex data, offering potential benefits in terms of predictive accuracy and versatility [17].

Second, this study aims to assay the active components, PYR, CYC, and MEC, in the presence of their related impurities. Third, the developed methods present a cheap and simple alternative to resolve complex mixtures rather than sophisticated chromatographic methods. Furthermore, this study considers environmental safety rules where ethanol is used as a solvent. Finally, it is a green solvent ranked after water to develop eco-friendly methods [18]. To achieve these goals, a five-level, five-factor calibration design was established to produce 25 mixtures for the calibration set. In addition, 16 experiments were performed for a test set distributed equally between the in-space and out-space samples. The developed models were tested to assay the studied drugs in the designed mixtures, and statistical tests were performed to compare them.

## 2. Results and Discussion

### 2.1. Optimization Parameters

Optimization of methods’ parameters was the first step to running models properly. For proper construction of OSC/CLS, DOSC/CLS, and NAP/CLS models, the number of projection matrix factors (for NAP/CLS) and the number of extracted factors (for OSC/CLS and DOSC/CLS) were optimized. For this reason, CV was applied where log PRESS values were calculated.

The details were as follows: five factors were needed for predicting PYR for building OSC/CLS and NAP/CLS models, while seven factors was the optimal number for the DOSC/CLS model. Regarding CYC, nine factors were needed for DOSC/CLS and NAP/CLS models, while OSC/CLS was built using eight factors. For building the proper models for MEC quantitation, eight factors were needed for OSC/CLS and NAP/CLS models, while nine factors were required for DOSC/CLS construction. It was clear that, as a general rule, DOSC/CLS always required a higher number of factors, which means that this model is more complicated than the others.

### 2.2. Data Analysis Results and Discussion

The presented work compares different augmented CLS chemometric models via analysis of different PYR, CYC, and MEC mixtures in the presence of two related impurities (BEH and BEP) as a case study.

**Figure 2:**
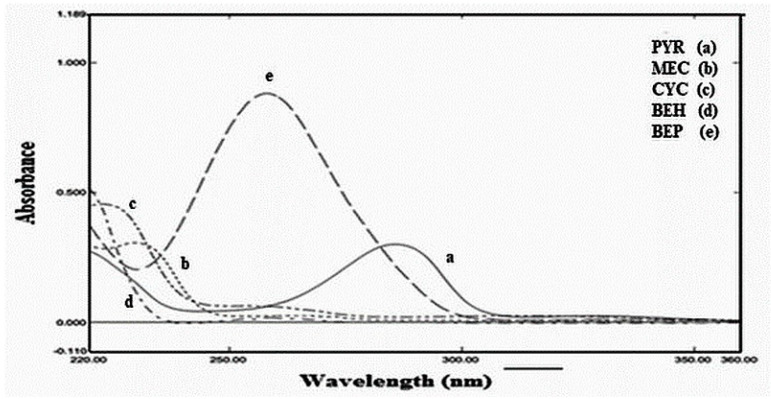
Superimposed UV spectra of (10 µg mL^−1^) of PYR (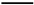), CYC ( _.._.._ ), MEC (…….), BEH ( _._._._._ ), and BEP (------) in absolute ethanol.

The structural similarity of the studied components makes their UV spectra strongly overlap, as shown in Figure 2.

This overlapping hinders their analysis by the classical univariate procedures and encourages the adoption of multivariate perspectives to assay this mixture. Additionally, the main goal of the presented study is to evaluate different augmented CLS models. In addition to the standard CLS one, the extended models were exploited for quantitation of the three analytes in the quinary mixture sets, as depicted in Table 1 and Table 2. For assessment of the models’ predictability on the calibration and validation sets, RMSEC and RMSEP were calculated, respectively. RMSEC and RMSEP were determined on the very same principle for training and test sets, respectively, according to the given equation.

(1) $$\mathrm{RMSEC}(P)=\sqrt{\frac{1}{I}\sum_{i=1}^{I}{\left(c_{i}-{\hat{c}}_{i}^{A}\right)}^{2}}$$

where I is the number of samples in the test set (in case of RMSEP) and I − 1 for the training set (in case of RMSEC), c_i_ is the known concentration for sample i, and ${\hat{c}}_{i}^{A}$ is the predictable concentration of sample i using A components.

**Figure 3:**
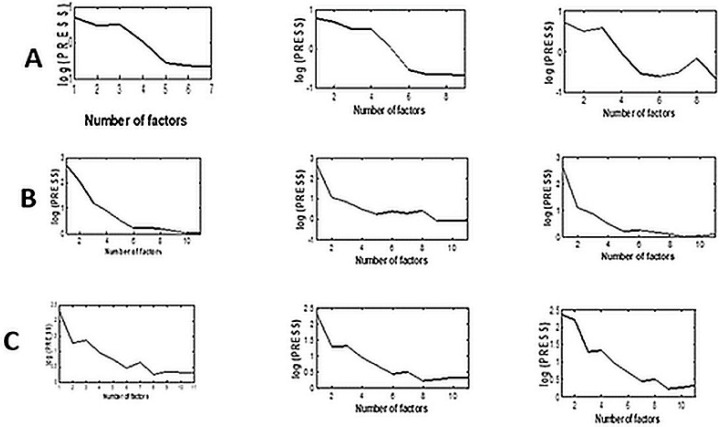
Selection of the optimum number of factors via plotting log predicted residual error sum of squares (PRESS) versus the number of factors using the cross-validation technique for (**A**) PYR, (**B**) CYC, and (**C**) MEC by OSC/CLS, DOSC/CLS, and NAP/CLS models, respectively.

Auto-prediction results (for the training set) are shown in Table 1. A recovery percentage of around 100% was obtained utilizing the four adopted models for the three analytes’ quantitation. However, the RMSEC values showed the superiority of the accuracy of the three augmented models over the classical CLS, especially for CYC and MEC prediction. Additionally, the three augmented models had higher precision than the classical ones, as indicated by their low S.D. values. Regarding the validation set (composed of 16 samples), it was designed in such a manner that eight samples intentionally lay outside the mixing area of the training set to evaluate the performance of the tested augmented models to predict future unplanned samples. Accordingly, we classified the validation set into in-space samples and out-space samples. The prediction results for the validation set are presented in Table 2. All models showed good performance predicting PYR concentrations in in-space and out-space samples. This may be attributed to the small overlap between all the components’ spectra of the quinary mixture with PYR, especially above 280 nm. The model performance discrimination occurred in CYC and MEC, where CLS showed low predictability compared to the other three augmented models. The three tested augmented CLS showed high and reproducible performance in CYC and MEC quantitation for in-space and out-space samples. Figure 3 shows the RMSEP plots for predicting the out-samples in the validation set for the three analytes using the four models, showing the high predictability in addition to the generalization (ability to predict unplanned samples) of the three augmented models. Furthermore, the augmented CLS models are more robust than CLS, which is indicated by acceptable recoveries and RMSEP values in Table 2, because they can accurately predict the out-of-space experiments that provide insights into the model’s applicability to unseen data and its potential for broader analytical use. Therefore, these findings deliver a powerful message to the quality control department that they could rely on augmented CLS models to assay unplanned samples.

### 2.3. Instrumental Consideration

The instrumental consistency during the whole study was validated using the PLS methodology to track the difference in the PYR concentration of one of the samples at six different analysis phases. The R% ± S.D. was 99.31% ± 0.43, implying that the significance of the instrumental deviation to the spectral results collected was lower than the replication variability.

### 2.4. Statistical Comparison to Reference HPLC Method

The suggested methods were then applied to analyze the available dosage forms (Table 3). The results showed good performance for the three models for analyzing the three analytes except for NAP/CLS in predicting MEC, where the recovery percentage was about 89.46%. Both the British Pharmacopoeia [1] and the International Conference on Harmonization (ICH) [19] state that the pharmaceutical dosage form acceptable recovery ranged from 95% to 105%. However, on further assessment of the data by comparing them statistically with the reported HPLC method [20], it was discovered that the only model that showed no significant difference with the HPLC method was the DOSC/CLS model regarding accuracy and precision (Table 3). The calculated t and F values were always less than the tabulated ones for the DOSC/CLS model for all the analytes, while for the other models, these values were bigger than the tabulated ones in some cases. Accordingly, DOSC/CLS was recommended to be the most accurate and reliable model among the tested ones, even though the OSC/CLS and NAP/CLS are of accepted accuracy and precision.

### 2.5. Figures of Merit

The figures of merit for the proposed enhanced CLS models were determined using the MVC1 toolbox. The findings are seen in Table 4. The best results were acquired from implementing the DOSC/CLS model. This is demonstrated by increased sensitivity, analytical sensitivity, and selectivity. Sensitivity tests the difference in responsiveness as a function of the analyte concentration, and analytical sensitivity is computed by dividing the sensitivity by instrumental noise. At the same time, selectivity implies the portion of the overall signal that is not missing due to spectral interference.

These findings proved the ability of the DOSC/CLS to remove the noise from the spectral data and retrieve pertinent information.

## 3. Materials and Methods

### 3.1. Instrument

A UV 1800 double-beam spectrophotometer (Shimadzu, Japan) was used. It was operated with UVProbe software, version 2.34. UV scanning was performed at 2 nm bandwidth and 2800/min speed.

### 3.2. Samples

Pure samples of PYR and MEC were gifts from Sigma Pharmaceuticals Industries (El Monofeya, Egypt), while CYC was granted from Amoun Pharmaceutical Co. (El Obour City, Cairo, Egypt). The purity of the PYR and MEC was 100.66 and 100.03%, respectively, according to the reported HPLC method [20], while the purity of CYC was 99.09%, as confirmed by another HPLC method [8]. BEH and BEP with 99% certified purity were purchased from Acros Organics chemical company.

### 3.3. Pharmaceutical Formulations

Sigma Pharmaceutical Co. was the source of Dizirest B6 tablets (Batch No. 33053). Each tablet should contain 50 mg of PYR and 25 mg of MEC. Emetrex tablets (Batch No. 151307) were produced by Amoun Pharmaceutical Co., with the claimed amount of 30 mg PYR and 50 mg CYC.

### 3.4. Solvents

Ethanol of HPLC grade was purchased from Sigma–Aldrich Chemie GmbH, Germany.

### 3.5. Standard Solutions

A quantity of 100 mg of PYR, CYC, and MEC was dissolved separately into three independent 100 mL volumetric flasks using ethanol to prepare 1000 µg mL^−1^ stock standard solutions. Stock standard solutions of BEP and BEH (100 µg mL^−1^) were prepared by dissolving 10 mg of each into two discrete 100 mL volumetric flasks using the same solvent.

Appropriate dilutions using ethanol were performed to prepare the working standard solutions with 100 µg mL^−1^ for PYR, CYC, and MEC and 10 µg mL^−1^ for BEP and BEH

## 4. Procedures

### 4.1. Linearity

Absorption spectra of the studied main drugs ranging from 1 to 60 µg mL^−1^ were recorded over the range of 221–370 nm [21]. The linearity of PYR and CYC was demonstrated between 3 and 40 µg mL^−1^ at their λ_max_ of 290 nm and 225 nm, respectively. MEC exhibits linearity between 4 and 55 µg mL^−1^ at its λ_max_ of 230 nm. The selected wavelength range was 221–370 nm, where the mixture under study shows no absorbance above 370 nm, and the solvent and product excipients may interfere below 221 nm [21]. The superimposed UV spectra of 10 µg mL^−1^ of all constituents are evident in Figure 2, showing intense overlap.

### 4.2. Experimental Design

#### 4.2.1. Calibration Set

**Figure 4:**
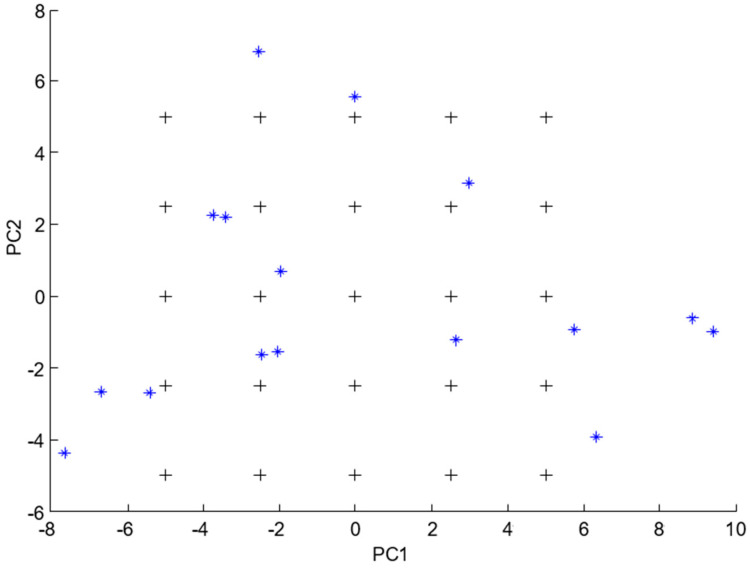
Scores plot for the mean-centered 25 samples of the training set (plus symbol), and 16 test set samples (asterisk symbol) indicate the in-space and out-of-space samples.

A five-level, five-factor calibration design was performed using five concentration levels with codes of (0, 1, 2, −1, and −2) while making the central level for each of the five components coded as zero. The central level was 10 µg mL^−1^ for PYR and MEC and 8.5 µg mL^−1^ for CYC. The design was planned to cover the mixture space sufficiently. The training set is composed of 25 mixtures, where there are five mixtures at each concentration level for each component [22,23]. Different aspects should be taken into consideration when determining the concentration for each level for a specific compound, namely, the calibration range of the three main drugs, the proportion of the studied drugs in the marketed dosage forms, and the percentage of the related impurities which must not exceed 5% of the main drugs relative to molar basis as shown in Table 5. The 2D scores plot of the first two P.C.s was attained using the mean-centered concentration matrix (Figure 4). Finally, the plot proved that all mixtures in the space were symmetric, orthogonal, and rotatable [22,23].

#### 4.2.2. Validation/Test Set

The predictive power of the studied chemometric models was tested by constructing an independent test set. The test set consisted of eight mixtures; four were regenerated from the training set (mixtures number 2, 6, 10, and 20). The other four mixtures were independently prepared within the concentration space of the design, as evident in Table 6 and Figure 4.

The generalization ability—the capability of the proposed models to predict unplanned samples—was tested by preparation of an extra eight samples, which are made to lie deliberately outside the mixture space, as shown in Figure 4.

### 4.3. Assay of Pharmaceutical Formulations

Twenty tablets of each of Emetrex^®^ and Dizerest B6^®^ tablets were separately powdered and mixed well; then, 0.07 and 0.128 gm from powdered Emetrex^®^ (equivalent to 10 mg CYC) or Dizerest B6^®^ (equivalent to 10 mg MEC) tablets, respectively, were transferred into two 100 mL volumetric flasks. A quantity of 50 mL of ethanol was added to each flask, and then the powder was ultrasonicated for 30 min. After cooling, the volume of each flask was completed with ethanol to obtain 100 µg mL^−1^ of CYC or MEC, and then the solutions were filtered. Quantities of 2.5 and 1 mL from Emetrex^®^ or Dizerest B6^®^ filtered solutions were separately transferred in two 10 mL volumetric flasks, and then the volume was completed using ethanol. The diluted solutions were scanned over the range of 221–370 nm three times, and then the average of the corresponding spectra was recorded. The experiment for each pharmaceutical formulation was repeated six times, and the proposed chemometric models analyzed the resultant spectra to determine the concentrations of PYR, CYC, and MEC.

### 4.4. Instrumental Stability

The instrumental stability was monitored over the analysis time by preparing a large volume—to be enough for analysis—of one of the training set mixtures (number 6 was chosen). The solution was stored in sealed vials at low temperatures and scanned every five measurements.

### 4.5. Software

Augmented CLS models were applied in Matlab^®^ 7.1.0.246 (R14) using MVC1 toolboxes [23]. The *t*-test and F-test were performed using Microsoft^®^ Excel 2019. The code for the classical CLS model was written by I. A. Naguib using Matlab in the lab.

### 4.6. Augmented CLS Models

CLS is a straightforward multivariate calibration technique that necessitates a detailed understanding of all components of the calibration matrix. Nevertheless, on the other hand, approaches such as partial least squares (PLS) may be used in quantitative analysis when one or more components remain unknown. This feature gave an advantage to the PLS over the CLS methodology [24]. The predictive capability of the conventional CLS model could be enhanced enormously by using various pre-processing procedures, including the NAP, OSC, and DOSC. Pre-processing data before the calibration stage could mitigate the impact of systematic alterations unrelated to the parameters of interest [25].

Description and theoretical overview are explored in depth in the literature for the CLS model [24], NAP/CLS [26], OSC/CLS [27], and DOSC/CLS) [28]. The tested augmented models presented in the current study are more straightforward than PLS because they are based on the well-known CLS approach.

Optimization of several factors for the modified CLS models.

To optimize the number of factors for building the tested methods [24], leave-one-out cross-validation (LOO-CV) was used via building the model using the *i* − 1 samples set (24 mixtures from the calibration set) to predict the left sample (validation sample). The PRESS (predicted residual error sum of squares) is computed as follows:

(2) $$PRESS=\sum_{i=1}^{i=N}{\left(\hat{C_{i}}-C_{i}\right)}^{2}$$

where *i* refers to the number of samples in the calibration set, $C_{i}$ is the known concentration for sample *i*, and $\hat{C_{i}}$ is the predicted concentration of the sample. Consequently, log PRESS values were plotted versus the number of factors (Figure 4) for choosing the optimal number of factors following Haaland and Thomas [29].

## 5. Conclusions

Different augmented CLS models have been applied for resolving the quinary mixture of the three analytes (PYR, CYC, and MEC) with two related impurities (BEH and BEP) in raw materials and pharmaceutical formulations.

These methods are OSC/CLS, DOSC/CLS, and NAP/CLS, in addition to the classical CLS model. The proposed models have qualitative power (estimation of pure spectra) as well as quantitative power (prediction of concentrations of the three analytes in different mixtures with the two related impurities). The developed methods are more rapid and straightforward than traditional spectrometric methods and other important analytical merits, such as sensitivity and selectivity. Among the proposed models, DOSC/CLS was the most powerful model with excellent quantitative power in addition to the well-known qualitative power of the CLS-based models. Only the DOSC/CLS model was successfully applied to determine the three analytes in the two dosage forms in all cases (Emetrex^®^ and Dizirest ^®^ tablets) without prior separation or interference from commonly encountered additives. Lastly, DOSC/CLS showed the best figures of merit, including sensitivity, analytical sensitivity, and selectivity.

## Figures and Tables

**Table 1:** Analysis results for predicting the training set (auto-prediction) of PYR, CYC, and MEC by the CLS and the augmented CLS methods.

PYR					CYC					MEC				
Training Set	CLS	OSC/CLS	DOSC/CLS	NAP/CLS	Training Set	CLS	OSC/CLS	DOSC/CLS	NAP/CLS	Training Set	CLS	OSC/CLS	DOSC/CLS	NAPCLS
Taken (µg/mL)	% R	%R	%R	%R	Taken (µg/mL)	% R	%R	%R	%R	Taken (µg/mL)	% R	%R	%R	%R
10.00	98.61	100.41	100.42	100.73	8.50	104.93	97.71	97.69	98.95	10	98.47	100.27	100.29	99.72
10.00	99.66	99.65	99.48	99.61	4.50	102.93	98.89	98.91	98.68	5	109.05	101.61	101.57	102.98
5.00	99.53	99.8	98.72	99.90	4.50	110.83	101.91	101.96	99.19	15	99.12	99.03	98.99	100.13
5.00	98.44	98.17	98.90	97.94	12.50	105.37	100.05	100.04	100.37	7.5	90.18	100.31	100.34	100.39
15.00	99.63	99.93	100.29	100.01	6.50	108.98	100.80	100.85	101.82	15	93.16	100.91	100.93	100.97
7.50	98.03	99.44	99.76	99.76	12.50	102.08	101.13	101.09	100.50	10	96.36	98.82	98.86	98.09
15.00	99.32	99.91	99.99	100.06	8.50	102.87	102.12	102.08	98.76	7.5	94.96	97.96	97.98	97.88
10.00	98.52	98.87	99.07	99.08	6.50	97.94	101.51	101.47	99.54	7.5	100.8	98.19	98.19	99.86
7.50	100.07	99.7	100.35	99.89	6.50	97.82	101.52	101.43	102.26	12.5	102.28	101.22	101.24	101.64
7.50	98.91	99.46	99.79	99.66	10.50	95.33	99.52	99.60	100.50	15	98.57	98.68	98.67	99.36
12.50	99.41	99.74	99.88	99.86	12.50	98.25	100.50	100.53	99.89	12.5	99.31	100.73	100.71	101.60
15.00	99.4	99.27	99.75	99.29	10.50	96.94	99.43	99.46	98.62	10	93.66	98.84	98.86	99.79
12.50	100.46	99.14	99.18	98.86	8.50	100.37	96.18	96.14	99.55	15	98.84	99.88	99.89	99.95
10.00	101.42	101.18	100.59	101.08	12.50	101.57	99.49	99.47	99.73	15	101.83	101.48	101.45	101.61
15.00	100.74	100.24	99.99	100.01	12.50	101.13	100.02	100.03	100.26	5	106.78	101.27	101.23	100.90
15.00	100.31	99.96	99.76	99.89	4.50	104.27	103.36	103.30	97.96	12.5	101.51	100.82	100.82	99.98
5.00	104.48	101.59	101.34	100.72	10.50	102.4	101.65	101.70	99.30	5	100.5	102.41	102.36	103.01
12.50	101.67	101.3	101.25	101.21	4.50	100.19	103.56	103.65	102.34	10	103.54	100.50	100.49	100.41
5.00	103.51	100.85	100.69	100.22	8.50	100.26	101.98	101.95	99.63	12.5	102.64	98.58	98.61	97.22
10.00	100.15	101.14	100.75	101.32	10.50	95.35	100.25	100.24	99.80	12.5	110.27	101.25	101.26	99.35
12.50	99.71	99.65	99.15	99.57	10.50	99.68	98.96	98.94	101.67	7.5	98.89	95.23	95.20	95.24
12.50	101.13	101	100.80	100.94	6.50	96.45	98.75	98.77	101.05	5	102.81	97.94	97.93	97.10
7.50	98.72	98.49	98.80	98.56	4.50	83.91	95.69	95.71	101.66	7.5	104.28	99.62	99.66	98.86
5.00	100.34	100.07	100.38	100.23	6.50	89.21	96.98	96.98	98.23	10	101.13	100.02	100.04	99.22
7.50	100.23	100.93	100.92	101.20	8.50	94.14	99.03	99.03	100.05	5	109.1	106.47	106.44	107.65
Mean %	100.10	99.99	100.00	99.98		99.73	100.04	100.04	100.01		100.72	100.08	100.08	100.12
SD	1.50	0.89	0.75	0.85		5.72	2.03	2.03	1.23		4.97	2.05	2.05	2.38
RMSEC	0.109	0.078	0.066	0.077		0.374	0.134	0.134	0.084		0.454	0.151	0.151	0.170

**Table 2:** Analysis results for predicting the validation set of PYR, CYC, and MEC by the CLS and the augmented CLS methods.

PYR					CYC					MEC				
Validation Set	CLS	OSC/CLS	DOSC/CLS	NAP/CLS	Training Set	CLS	OSC/CLS	DOSC/CLS	NAP/CLS	Training Set	CLS	OSC/CLS	DOSC/CLS	NAP/CLS
Taken (µg/mL)	% R	%R	%R	%R	Taken (µg/mL)	% R	%R	%R	%R	Taken (µg/mL)	% R	%R	%R	%R
**In Space**														
7.00	97.82	94.29	93.47	93.41	12	108.42	100.86	100.73	100.29	10	91.34	96.46	96.46	96.52
8.00	95.58	92.50	92.07	91.81	8	107.41	97.26	97.12	94.33	10	90.30	96.02	96.01	96.79
14.00	98.07	96.14	95.97	95.58	10	111.87	98.49	98.46	97.57	10	81.68	95.21	95.18	96.68
8.00	96.86	95.25	94.40	94.96	6	109.02	100.21	100.00	95.00	12	99.08	99.02	99.00	99.22
10.00	99.66	99.60	99.48	99.61	4.5	102.93	98.89	98.91	98.68	5	109.05	101.61	101.57	102.98
7.50	98.03	99.47	99.76	99.76	12.5	102.08	101.13	101.09	100.50	10	96.36	98.82	98.86	98.09
7.50	98.91	99.47	99.79	99.65	10.5	95.33	99.52	99.60	100.50	15	98.57	98.68	98.67	99.36
10.00	100.15	101.10	100.75	101.32	10.5	95.35	100.25	100.24	99.80	12.5	110.27	101.25	101.26	99.35
Mean %	98.14	97.22	96.96	97.01		104.05	99.58	99.52	98.33		97.08	98.38	98.38	98.62
SD	1.48	3.09	3.38	3.51		6.25	1.31	1.31	2.48		9.56	2.35	2.35	2.15
**Out Space**														
8.00	96.46	94.5	93.85	94.06	10	104.70	99.07	98.94	97.42	5	97.40	98.11	98.08	99.54
5.00	95.47	93.6	92.06	93.15	10	102.74	99.80	99.66	95.41	5	105.46	98.02	98.01	97.27
18.50	98.65	96.86	96.77	96.37	5.5	122.33	101.49	101.47	95.88	6	71.70	93.10	93.08	93.89
16.00	98.81	97.19	96.93	96.73	8	110.84	99.33	99.25	96.43	8	84.95	93.79	93.79	93.43
5.00	93.65	88.4	89.89	87.62	7	100.19	93.59	93.47	92.59	17	91.59	99.03	99.06	99.29
17.00	97.18	95.59	95.56	95.17	5	120.30	101.77	101.70	93.87	10	79.59	94.85	94.87	94.18
18.00	98.85	97.28	97.22	96.84	6	115.74	100.09	100.10	95.92	6	71.32	93.61	93.60	94.20
5.00	96.24	93.6	93.99	93.34	7	95.88	96.37	96.22	95.94	15	98.14	97.64	97.67	97.12
Mean %	96.91	94.63	94.53	94.16		109.09	98.94	98.85	95.43		87.52	96.02	96.02	96.11
SD	1.85	2.94	2.60	3.03		9.72	2.72	2.76	1.52		12.71	2.41	2.42	2.50
RMSEP	0.283	0.509	0.520	0.566		0.755	0.191	0.197	0.338		1.307	0.354	0.353	0.369

**Table 3:** Statistical comparison of the results obtained by the proposed CLS-based chemometric methods and reported HPLC method on Emetrex^®^ and Dizerest B6^®^ tablets.

Parameters		CLS		OSC-CLS		DOSC-CLS		NAP-CLS		Reported HPLC Method [7] ^a^	
		PYR	CYC	PYR	CYC	PYR	CYC	PYR	CYC	PYR	CYC
Emetrex^®^ tablets (B.N.151307)	Mean %	97.91	97.62	99.60	103.64	99.25	103.69	99.81	101.69	100.13	105.60
	SD	1.62	0.59	1.53	0.61	1.58	0.58	1.46	1.27	1.86	0.98
	n	6	6	6	6	6	6	6	6	6	6
	Student’s *t*-test	−2.200 (2.228) *	11.682 (2.228) *	0.736 (2.228) *	−1.663 (2.228) *	0.876 (2.228) *	−1.785 (2.228) *	0.330 (2.228) *	1.856 (2.228) *	-------	-------
	*F*-value	1.320 (5.050) *	2.548 (5.050) *	1.472 (5.050) *	2.335 (5.050) *	1.396 (5.050) *	2.605 (5.050) *	1.625 (5.050) *	1.837 (5.050) *	--------	-------
Dizerest B6^®^ tablets (B.N. 33053)		CLS		OSC-CLS		DOSC-CLS		NAP-CLS		Reported HPLC method [20] ^b^	
		PYR	MEC	PYR	MEC	PYR	MEC	PYR	MEC	PYR	MEC
	Mean %	100.36	106.72	102.75	97.86	101.18	97.96	103.37	89.46	98.80	98.67
	SD	1.37	0.87	1.37	0.64	1.28	0.63	1.34	1.21	1.72	1.38
	n	6	6	6	6	6	6	6	6	6	6
	Student’s *t*-test	−0.632 (2.228) *	−12.092 (2.228) *	−3.300 (2.228) *	1.308 (2.228) *	−1.585 (2.228) *	1.149 (2.228) *	−4.011 (2.228) *	12.30 (2.228) *	--------	-------
	*F*-value	1.568 (5.050) *	2.501 (5.050) *	1.575 (5.050) *	4.643 (5.050) *	1.781 (5.050) *	4.805 (5.050) *	1.625 (5.050) *	1.299 (5.050) *	--------	-------

* Figures in parentheses represent the corresponding tabulated values of t and *F* at *p* = 0.05. ^a^ The mobile phase consisted of acetonitrile: 0.05M KH_2_PO_4_ in a ratio of 50:50 (*v*/*v*). The pH was adjusted to 4.0 with phosphoric acid; the separation was carried out on a (25 cm × 4.6 mm, 5 μm) RP C18 column. The UV detector was adjusted at 239 nm. ^b^ The mobile phase consisted of NaH_2_PO_4_ buffer: acetonitrile: trifluoroacetic acid at a 30:70:0.1 ratio by volume. The separation was carried out on a (25 cm × 4.6 mm, 5 μm) RP C18 column, and the UV detector was adjusted at 254 nm.

**Table 4:** Calculated figures of merit for the three analytes using the augmented CLS methods.

Figures of Merit	PYR			CYC			MEC		
	OSC/CLS	DOSC/CLS	NAP/CLS	OSC/CLS	DOSC/CLS	NAP/CLS	OSC/CLS	DOSC/CLS	NAP/CLS
Sensitivity ^a^	0.071	0.15	0.067	0.023	0.11	0.006	0.014	0.12	0.011
Analytical sensitivity ^b^	54	110	48	62	220	17	39	270	29
Selectivity ^c^	0.46	1	0.44	0.21	1	0.055	0.12	1	0.086

^a^ Sensitivity measures the changes in response as a function of the concentration of a particular analyte. ^b^ Analytical sensitivity equals sensitivity divided by instrumental noise. ^c^ Selectivity indicates the part of the total signal that is not lost due to spectral overlap.

**Table 5:** The 5-level 5-factor experimental design of 25 training mixtures is shown as concentrations of the mixture in µg/mL.

NO	PYR	CYC	MEC	BEP	BEH	NO	PYR	CYC	MEC	BEP	BEH
1	10	8.5	10	0.25	0.25	14	10	12.5	15	0.15	0.3
2 *	10	4.5	5	0.35	0.2	15	15	12.5	5	0.3	0.15
3	5	4.5	15	0.2	0.35	16	15	4.5	12.5	0.15	0.25
4	5	12.5	7.5	0.35	0.25	17	5	10.5	5	0.25	0.3
5	15	6.5	15	0.25	0.2	18	12.5	4.5	10	0.3	0.3
6 *	7.5	12.5	10	0.2	0.2	19	5	8.5	12.5	0.3	0.2
7 *	15	8.5	7.5	0.2	0.3	20	10	10.5	12.5	0.2	0.15
8	10	6.5	7.5	0.3	0.35	21	12.5	10.5	7.5	0.15	0.2
9	7.5	6.5	12.5	0.35	0.3	22	12.5	6.5	5	0.2	0.25
10	7.5	10.5	15	0.3	0.25	23	7.5	4.5	7.5	0.25	0.15
11	12.5	12.5	12.5	0.25	0.35	24	5	6.5	10	0.15	0.15
12	15	10.5	10	0.35	0.35	25	7.5	8.5	5	0.15	0.35
13	12.5	8.5	15	0.35	0.15						

* represents the ratio of pharmaceutical formulations.

**Table 6:** Sixteen test set mixtures indicate the in-space and out-of-space samples shown as concentrations of the mixture in µg/mL.

NO	PYR	CYC	MEC	BEP	BEH	Position
1	7.00	12.00	10.00	0.20	0.20	IN
2	8.00	8.00	10.00	0.25	0.25	IN
3	14.00	10.00	10.00	0.35	0.25	IN
4	8.00	6.00	12.00	0.15	0.25	IN
5	10.00	4.50	5.00	0.35	0.20	IN
6	7.50	12.50	10.00	0.20	0.20	IN
7	7.50	10.50	15.00	0.30	0.25	IN
8	10.00	10.50	12.50	0.20	0.15	IN
9	8.00	10.00	5.00	0.25	0.30	OUT
10	5.00	10.00	5.00	0.15	0.25	OUT
11	18.50	5.50	6.00	0.30	0.20	OUT
12	16.00	8.00	8.00	0.25	0.25	OUT
13	5.00	7.00	17.00	0.30	0.20	OUT
14	17.00	5.00	10.00	0.15	0.25	OUT
15	18.00	6.00	6.00	0.25	0.30	OUT
16	5.00	7.00	15.00	0.30	0.20	OUT

## Data Availability

Datasets can be provided by authors upon request.
